# Sequencing to identify pathogens in *Tenebrio molitor*: Implications in insects farmed for food and feed

**DOI:** 10.3389/finsc.2023.1059046

**Published:** 2023-01-30

**Authors:** Dewey Leierer, Morgan Olmstead, Brenda Oppert

**Affiliations:** ^1^ Department of Biochemistry and Molecular Biophysics, Kansas State University, Manhattan, KS, United States; ^2^ United States Department of Agriculture Agricultural (USDA) Research Service, Center for Grain and Animal Health Research, Manhattan, KS, United States

**Keywords:** *Tenebrio molitor*, yellow mealworm, insects for food, insect pathogens, shotgun metagenomics, farmed insects

## Abstract

**Introduction:**

The farmed insect industry is increasing in number and size to meet the demand for sustainably-produced protein. Larger insect farms are prone to losses due to pathogens, and more information is needed regarding the health of insects reared for food and feed.

**Methods:**

In this study, high throughput sequencing was used to identify potential pathogens in a colony of *Tenebrio molitor* (yellow mealworm, Coleoptera: Tenebrionidae) that exhibited increased mortality in immature stages with eventual colony collapse. Sequences also were obtained from a healthy new colony of *T. molitor*, as well as a recovered individual from the collapsed colony.

**Results:**

Screening of sequences obtained from the colonies and their rearing diet indicated that the collapsed colony had low diversity in microbial taxa, with predominantly sequences from the families Staphylococcaeceae and Streptococcaceae constituting from 53 to 88% of the total microbial reads. Conversely, in the new colony and their rearing diet, microbial sequences were from more than 15 different taxa, with Lactobacilleceae the most prevalent but representing only 21% of the total microbial reads. Evidence indicates that *Bacillus thuringiensis* may have been involved in the collapse of the colony, leading to sepsis and microbial dysbiosis, although the source of the bacteria was not identified. Sequences from the recovered individual reflected a microbial flora profile that was intermediate between those of the diseased collapsed and new colonies.

**Discussion:**

These findings have implications for insects reared in confined environments and provide a rapid method to screen insect colonies by sequencing healthy and potentially diseased individuals.

## 1 Introduction

The global population is growing exponentially, with current forecasts exceeding 9 billion people by 2050 (1). To feed a population of that size, annual food production must increase by 70% and annual meat production must increase by 200 million tons. Obstacles to increased food production include climate change, depleting water sources, and restrictions on expanding farmable land. In addition, animal feed production costs from protein-rich ingredients like fishmeal and soybean meal are increasing (2). Therefore, sustainable alternative protein sources are needed to supplement food and feed, and insects are promising candidates. Insects are high in nutrients like insoluble fiber, proteins, vitamins, and minerals (3). Insects also consume less water per gram of protein produced and require lower input feeds than most traditional livestock (4). The yellow mealworm, *Tenebrio molitor*, is one of the most popular and well-researched insects for supplementing animal feed. Companies already mass-rear *T. molitor* on an industrial scale for feeding domestic birds, fish, and reptiles (5).

There is considerable research on diseases affecting *T. molitor* because of its notoriety as a pest of poultry houses and its use as a lab model for coleopteran biochemical research. *Pseudomonas* and *Serratia* species are opportunistic bacterial pathogens that infect *T. molitor* colonies under stress (6, 7). Gregarines are known apicomplexan parasites of *T. molitor*, but infection typically only results in a decrease in the adult lifespan, not affecting the fitness of larval stages (8, 9). Entomopathogenic fungi, including those from the genera *Metarhizium* and *Beauveria* (10), as well as nematodes (11) also have been documented as potential biological control agents for *T. molitor*. Our research has used *T. molitor* as a model for studies of the mode of action of *Bacillus thuringiensis* Cry3Aa toxins in coleopterans (12–16).

Often insects in rearing facilities are in population-dense environments with a high potential for pathogen spread throughout the colony (17). Insects do not have an adaptive immune system, so disease prevention methods that rely on learned immunity, like vaccines, cannot be used to prevent illness in insects as they do with traditional livestock. Although some entomopathogens may be prevented or treated with antibiotics, overuse of antibiotics in other farming industries has contributed to the development of antibiotic-resistant bacteria (18). Farmed insects represent considerable investment, and as such there is increased interest in identifying diseases that may affect insects produced for feed, and development of prophylactics and treatments for insect diseases in reared systems (19, 20). Current prevention and treatment recommendations are suitable for smaller rearing facilities or for those who rear insects at home but are not practical for larger facilities (5). Our long-term goal is to assist larger facilities in avoiding the practice of removing infected colonies and replacing with new stock, especially with the costs of genetically-selected or modified strains. More research is needed on the detection, treatment, and prevention of pathogens that may affect mass-rearing efficiency and colony health of mealworms.

This case study uses DNA sequencing to identify pathogens that may be responsible for the collapse of a *T. molitor* colony that had decreased survival of egg to adult and subsequent mite infestation. The data identifies presumably beneficial bacteria in a healthy mealworm microbiome and pathogenic bacteria that were associated with the *T. molitor* colony collapse.

## 2 Methods

### 2.1 Colony rearing

*T. molitor* colonies were reared on a diet consisting of 90% organic Golden Buffalo™ whole wheat flour, 5% brewer’s yeast, and 5% stabilized wheat germ for more than 20 years. Slices of sweet potatoes were added to colonies to provide moisture and additional nutrients. Colonies were kept at 27 °C, 65 R.H in 2 L glass jars. When increased larval mortality was observed over time, the colony was moved to a separate chamber to be reared in quarantine (25 °C, 65 R.H), and a new colony was established in October, 2015 from commercial sources (reptilefood.com, vita-mealie.weebly.com, and rainbowmealworms.net) in the original chamber (27 °C, 65 R.H). The new colony was reared in ventilated plastic boxes, ranging in size: 19 x 12.5 x 8 cm (small larvae, 0-1 mo), 35 x 20 x 12 cm (medium larvae, 1-3 mo, and 39 x 34 x 8 cm (>3 mo, large larvae, pupae, adults). After the collapse of the original colony, the few surviving larvae of the quarantined colony (approximately 15) were placed in a 100 mL plastic cup with fresh diet. These larvae were kept in the lab at room temperature at ambient lab humidity.

### 2.2 DNA extraction and sequencing

DNA was extracted from samples including: samples from the collapsed colony - *T. molitor* adult (C-Adult), frass (C-Frass), and Diet (C-Diet); a recovered larva from a colony established from the remaining members of the collapsed colony (R-Larva); a pupa (N-Pupa) and adult (N-Adult) from a new colony and a diet sample (N-Diet) from the rearing container of the new colony containing both flour and frass (**Table 1**). At the time of sequencing, the original colony already was collapsing with few survivors. One dead adult was sequenced, the diet it had been reared on, and the frass from the colony. The remaining larvae (about 15) were placed on fresh diet to reduce mite infestation and to see if recovery was possible – one of the larvae (R-Larva) was sequenced. An extraction of DNA from a sample of pre-rearing diet (i.e., “fresh diet”, F-Diet) also was sequenced. Samples were stored at -20°C until extraction.

**Table 1:** Summary of sample type, description, and collection date.

Abbreviation	Sample	Description	Collection Date
C-Adult	Collapsed Colony Adult	Adult collected from colony at time of collapse (dead).	May 2021
C-Frass	Collapsed Colony Frass	Frass collected from colony at time of collapse.	May 2021
C-Diet	Collapsed Colony Diet	Flour substrate collected from collapsed colony at time of collapse.	May 2021
R-Larva	Recovered Larva	Larva collected from the recovered colony (living).	June 2021
N-Adult	New Colony Adult	Adult collected from the newly established colony (living).	January 2022
N-Diet	New Colony Diet	Rearing diet from the new colony	January 2022
N-Pupa	New Colony Pupa	Pupa collected from newly established colony (living).	January 2022
F-Diet	Fresh Diet	Sample of pre-rearing diet.	August 2022

Samples were ground in kit buffer (E.Z.N.A. Insect DNA kit, Omega Biotek, Norcross, GA, USA) with a micropestle in a 1.5 mL microcentrifuge tube and DNA was extracted using 4 μl RNase (instead of 2), an optional column equilibrium step, and an additional wash step prior to matrix drying, all variations from the recommended protocol. The extraction kit was optimized for extracting from samples with high polysaccharide content. Sequencing libraries were generated using an NGS Library Preparation Enzymatic Fragmentation kit (Twist Biosciences, CA, USA, version 2.0) following manufacturer’s protocol. Average library size was 460 bp for the Collapsed, Recovered, and New colony samples and 363 bp for the F-Diet sample. Sequencing was on a MiSeq (Illumina, San Diego, CA) with v3 600 cycle and v2 300 cycle nano kits, 151X151 bp paired end. Sequencing metrics are provided in **Table 2**.

**Table 2:** Sequencing metrics and classification of reads by Kraken2 (21).

Sample	Total reads	Total classified reads	Percent Classified reads
C-Adult	7,322,142	671,310	9.17%
C-Frass	7,584,608	2,163,158	28.52%
C-Diet	8,990,803	388,244	4.32%
R-Larva	4,514,734	93,404	2.07%
N-Adult	3,774,632	202,973	5.38%
N-Diet	9,253,710	348,601	5.75%
N-Pupa	4,459,590	531,933	7.82%
F-Diet	23,137,004	663,383	2.87%

### 2.3 Data analysis

Fastq files were converted to fasta files and were submitted to Kraken2 analysis (21) in Omicsbox (Biobam, Valencia, Spain, version 2.1.4 confidence interval filter of 0.4, RefSeq 2021.04). Classified reads from the superkingdom Bacteria were extracted into a separate file and resubmitted to Kraken2 for further analysis.

A bacterial species count table was exported from Omicsbox to compare the number of reads associated with each species across the collapsed, recovered, and new colony samples (**Supplementary File 1**). Reads from each sample were combined into categories (C-Adult, C-Frass, C-Diet in the collapsed colony and N-Adult, N-Diet, and N-Pupa in the new colony). Only species with counts over 100 reads in at least one colony group were used in the calculations. Classified microbial reads that were identified to the species level as *B. thuringiensis* were selected and analyzed by blastn at NCBI (accessed July 28, 2022, default parameters).

## 3 Results

**Figure 1:**
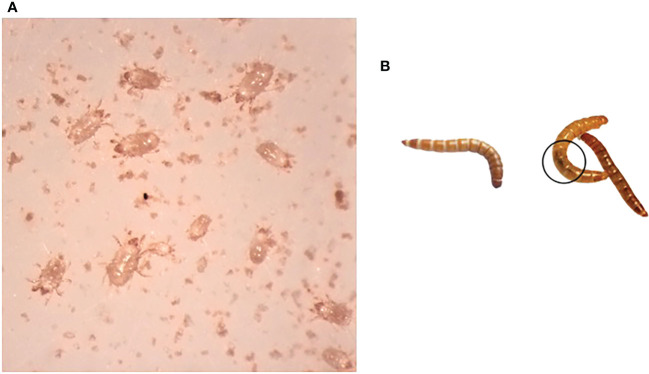
Observations of the collapsed colony of *T. molitor.* **(A)** Mites (unidentified) isolated from collapsed colony. **(B)** Larva from the newly established colony (left) and larvae recovered from the collapsed colony. Melanized gut is encircled.

This study investigated a *T. molitor* colony that demonstrated reduced viability and eventual collapse. In May 2021, the colony suffered a flour mite infestation that likely contributed to the collapse by competing for resources (**Figure 1A**), with only a few *T. molitor* larvae surviving. Larvae from the collapsed colony had reduced movement with darkening and melanization of the gut, evidence of bacterial infections in *T. molitor* (20, **Figure 1B**). When problems were identified with the original colony, *T. molitor* were obtained from three commercial sources and eventually combined into a colony labeled “new”. The approximately 15 surviving larvae from the collapsed colony were transferred to a new container with fresh diet and remained quarantined from the newly established colony. With careful monitoring, the survivors of the collapsed colony, labeled “recovered”, were able to pupate, reproduce, and began to reestablish. As time passed, there were fewer larvae exhibiting signs of infection, and the mite population was brought under control with careful hygiene.

**Figure 2:**
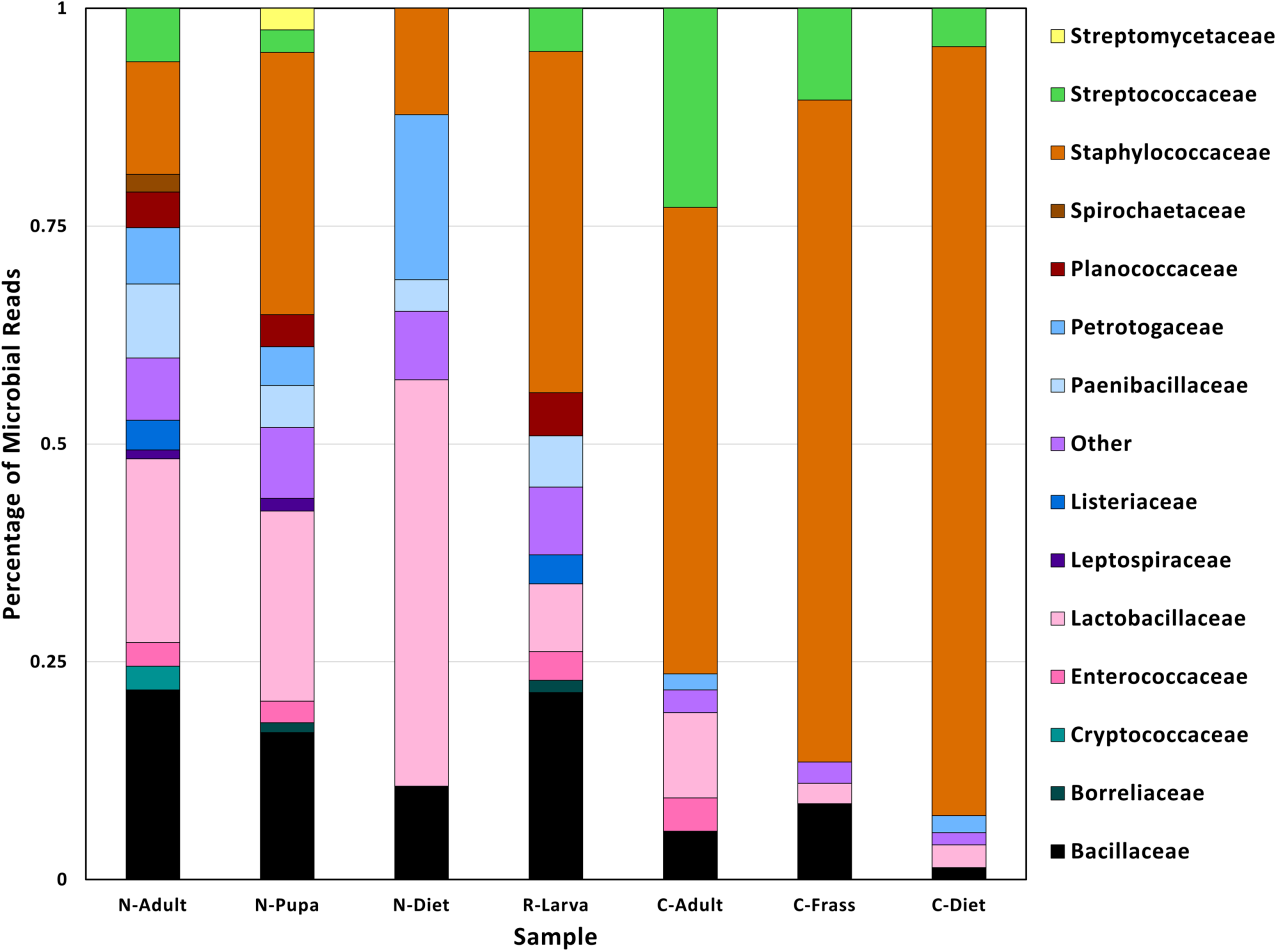
Family taxa found in different samples found in *T. molitor* DNA sequences. Abbreviations for samples are in **Table 1**.

We sequenced DNA from an adult from the collapsed colony (C-Adult), as well as their frass (C-Frass) and diet (C-Diet), and DNA from a recovered larva (R-Larva) (**Table 1**). We also sequenced DNA from a new colony adult (N-Adult) and their frass and diet combined (N-Diet). Sequencing provided from 3.8 to 9.3 million reads for downstream analysis (**Table 2**). Additionally, we sequenced over 23 million reads from fresh diet (F-Diet). Reads were analyzed for similarity to sequences in microbial databases. The majority of reads in all samples were unclassified, as they were likely host (*T. molitor*, **Additional File S1A**). At the superkingdom level, most reads were bacterial (**Additional File S1B**), the focus of this study. Diets had a higher percentage (16.4-29.3%) of eukaryotic reads than insect samples (0.99-9.93%) due mostly to contribution from reads similar to *Saccharomycetes* and likely due to the yeast in the diet (**Additional File S1A**). Some reads were assigned to *Mammalia*, but likely those were insect reads aligning to orthologs in the database. Fewer reads were associated with archea and viruses.

The lowest percentage of classified reads was from R-Larva DNA (2%), whereas 28% of reads from the C-Frass sample were classified (**Table 2**). We compared reads from the major bacterial classifications among the samples, focusing on of those were assigned to families at 1% or more of the total reads (**Figure 2**). Overall, the taxonomic distribution of the bacterial reads from the new colony samples were more similar, and those of the collapsed colony were similar. The taxonomic distribution of the bacterial reads from the R-Larva sample was intermediate to the distributions of those from the new colony and the collapsed colony samples.

Reads derived from the family Staphylococcaeceae were predominant in the microbial reads from the collapsed colony samples, constituting 53, 76, and 88% of the total classified reads in C-Adult, C-Frass, and C-Diet, respectively (**Figure 2**). In contrast, reads associated with Staphylococcaeceae in the new colony samples were 12, 13, and 30% for N-Diet, N-Adult, and N-Pupa, respectively. Reads from the bacterial family Streptococcaceae also were prevalent in the collapsed colony, constituting 4-23% of classified reads compared to 0-6% in the new colony. The newly established colony had higher percentages of bacteria from the families Lactobacillaceae (21-47%), Paenibacillacea*e* (4-9%), and Petrotogacea*e* (4-19%) than the collapsed colony (2-10, 0, 0-2%, respectively).

At the genus level, large increases in reads from the collapsed colony samples were observed from the genera *Mammaliicoccus*, *Lactococcus*, *Staphylococcus* compared to those from the new colony samples (**Additional File S2**). However, the collapsed colony samples had fewer reads from the genera *Lactobacillus* and *Lacticaseibacillus* than those from the new colony samples, in agreement with observations at the family level.

We were particularly interested in the family Bacillaceae since the phenotype of some of the collapsed insects suggested an active *B. thuringiensis* infection (**Figure 1**). Therefore, we examined sequences in the genus *Bacillus* through a targeted blast search to the *B. thuringiensis* database at NCBI. There were a larger number of reads from the Bacillaceae family in the samples from the new colony, but most of these were from different *Bacillus* species and other Bacillaceae genera (**Additional File S3**). More reads with high scoring blastn matches to *B. thuringiensis* were found in the collapsed colony samples, especially in the C-Frass sample (**Table 3**). The number of reads in the new colony samples were too low (1, 2) to confirm the presence of *B. thuringiensis*. However, 240 reads from C-Frass aligned to *Bacillus* spp, and 74 aligned to *B. thuringiensis* serovar morrisoni str. 4AA1 (taxid ID 1461024) with evalues < 10^-40^ (**Additional File S3**).

**Table 3:** Microbial reads from *T. molitor* samples that were similar to *B. thuringiensis* serovars, with more reads aligning to serovar *Morrisonii* in bold.

Sample	Number of reads aligned to *Bacillus thuringiensis*	Serovar (number of reads)
N-Pupa	1	None
N-Adult	3	*Alesti* (2)
N-Diet	8	*Monterrey* (1)
R-Larva	1	None
C-Adult	8	*Morrisoni* (4)
C-Diet	6	*Andoulensis* (1)
C-Frass	149	*Alesti* (6) *Andalousiensis* (8) *Huazhongensis* (1) *Kurstaki* (2) ***Morrisonii* (74)** *Pakistani* (24) *Pulsiensis* (2)
F-Diet	0

Fresh samples of rearing diet (F-Diet) were sequenced to confirm that was not the original source of *B. thuringiensis* in our collapsed *T. molitor* colony. The number of F-Diet sample reads were more than 4-fold greater than those in the other samples to ensure that *B. thuringiensis* would be identified if present (**Table 2**). However, none of the reads in F-Diet were identified as *B. thuringiensis*, indicating that the rearing diet likely was not the source of *B. thuringiensis* found in the other samples (**Table 3**).

**Figure 3:**
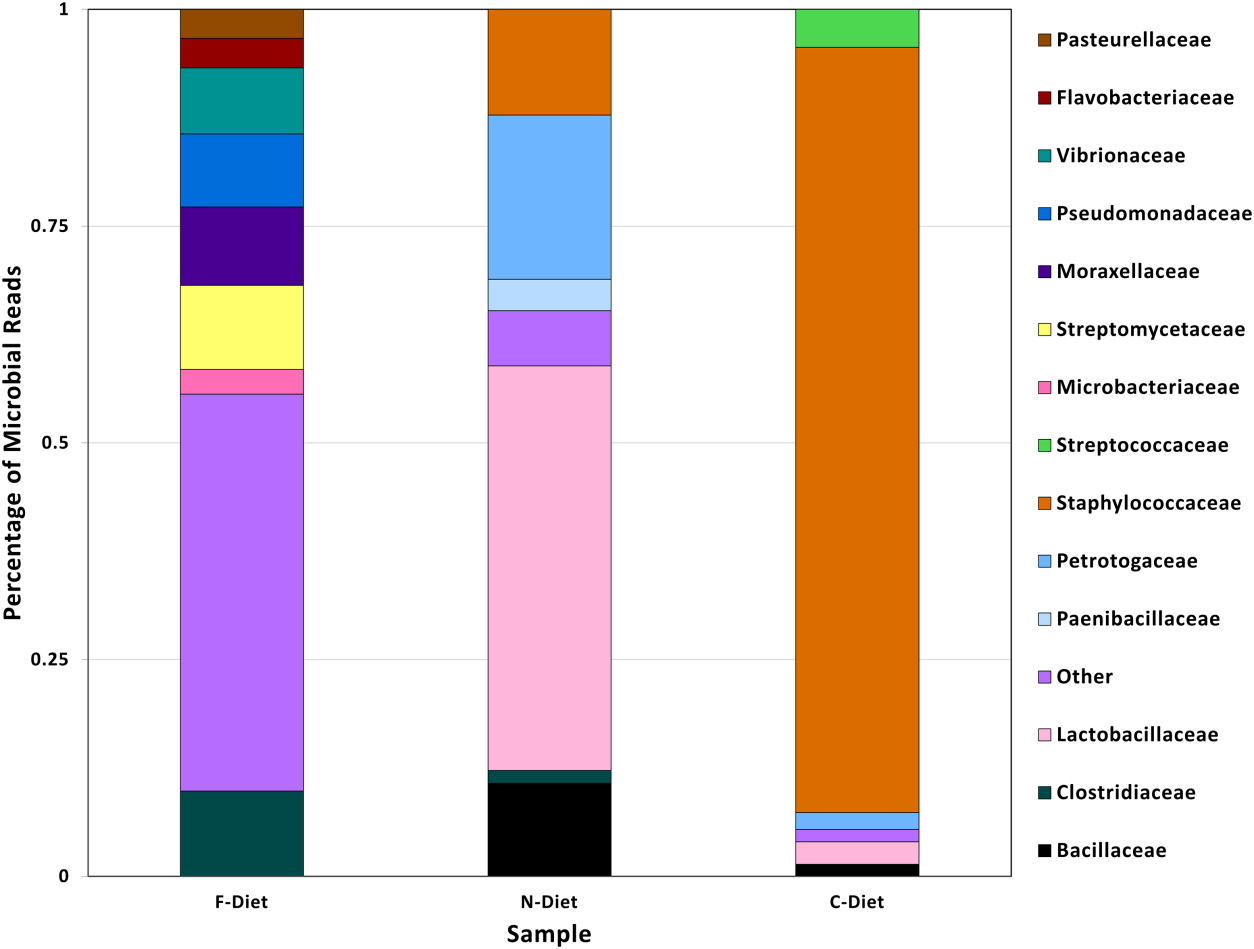
Comparison of bacterial taxa in sequences from pre-rearing diet (F-Diet) compared to diets from the new colony (N-Diet) and collapsed colony (C-Diet).

A comparison of microbial sequences from diets demonstrated the progression of loss of microbial diversity in pre-rearing diet to diet collected from the collapsed colony (**Figure 3**). The pre-rearing diet (F-Diet) only had 0.4% of microbial reads but from a more diverse bacteria flora, whereas the bacterial profile in diet from the new colony (N-Diet), also 0.4% of total reads, had shifted to primarily Lactobacilleceae. In contrast, microbial sequences in the diet from the collapsed colony (C-Diet), with 2.5% of the classified sequences as bacteria, had shifted to Staphylococceae with fewer other bacterial sequences found.

## 4 Discussion

High throughput sequencing can be an effective tool to understand the microbial profiles in insects, and this approach can be even more effective in diagnostics of diseased colonies or routine monitoring of farmed insects. Successful results are dependent on sequencing depth and the accuracy of microbial databases. We were limited by sample size in the collapsed colony, as only a few individuals survived. While we were able to identify meaningful and actionable data from the small number of samples in this study, improvements could be made with additional samples from different developmental stages and increased sequencing depth. As more insect and microbial sequences are acquired by data depositories, the accuracy of predicting microbial profiles will be improved.

In this study, we found that reads from the families Staphylococcaeceae and Streptococcaceae collectively constituted 76% of microbial families in the adult sequenced from the collapsed colony, with the bacterial communities of the frass and diet from that colony also containing large numbers of reads (93% and 86%, respectively, of the total bacterial reads in each sample). This narrow microbial diversity represented a shift from the more diverse community found in the adult from the new colony, with only 21% of bacteria from Lactobacilleceae, and the remainder of microbial reads spread over more than 15 different families. Similar profiles were found in the diet from the new colony. There also was a difference in the overall relative abundance of bacterial reads found in the two colonies, as the total number of microbial reads in the new colony was 62-68% for the sequenced larva and adult, whereas more microbial reads (85%) were found in the collapsed adult.

The phenotype of the diseased adult in the collapsed colony suggested there was an active *B. thuringiensis* infection, and our sequencing data support this observation. There were 74 reads from the collapsed colony diet that aligned to *B. thuringiensis* serovar *morrisoni*, and this serovar typically expresses Cry3Aa toxins that are insecticidal to Coleoptera (22, 23). However, it is unclear how the infection occurred, although finding a small number of reads aligning to *B. thuringiensis* in the new colony adult and diet suggests that these bacteria were present, possibly *via* the sweet potatoes that are introduced to larvae and adults during rearing.

These data do not inform us as to whether the bacteria identified in the collapsed adult contributed to the colony collapse, but more likely they were a result of the sepsis that occurs in the intestinal tract during infection with the insecticidal toxins from *B. thuringiensis. Staphylococcus aureus* was found in the intestinal tract of *Musca domesticus* and is speculated to be contained in that insect *via* excretion, digestion, and antimicrobial peptides, indicating that insects may have an active system to contain harmful bacteria (24). Streptococci, specifically enterococci, have been identified in more than half of insects surveyed, and it was speculated that insects may serve as an overwintering host for these bacteria (25). Given that the adult sequenced from the adult colony was dead, we cannot know if the microbiota populations were a result of postmortem changes in the insect. On the other hand, our data suggests that Lactobacilli may be beneficial to mealworms as they were found in 21% of the microbial reads from the healthy adult in the new colony. In Hymenoptera, evidence supports that hypothesis, in that some bees have host-specific Lactobacilli, and may be important for bee health (26). Lactobacilli can check the growth of other bacteria and are often components of probiotics, and these bacteria were among those suggested as beneficial for farmed insects (27). Our data supports the hypotheses that all of these bacteria are part of the normal flora of the mealworm gut tract and only become pathogenic when other factors disrupt their balance in the microbiome.

A healthy microbiome has a large diversity of species over highly diverged phylogenetic taxa (28). Divergent taxa fill unique roles within a system, but when closely related taxa increase in a biological system, these roles break down and a dysbiosis event occurs. Our data indicates that during the bottleneck event of the collapsed colony, a dysbiosis occurred where there was a major shift in the microbiome, either leading to the colony collapse or a factor of the collapse.

While the high mite infestation was competing for resources and emphasizes the need for frequent culturing of reared insects, we never found the mites directly feeding on *T. molitor* developmental stages. There are no references that indicate that mites can pass on pathogens to insects, but that possibility remains.

More data are needed on the natural and diseased profiles of insects reared for downstream food applications. Our efforts are in developing insect-specific pathogen databases that can rapidly and accurately identify insect pathogens harmful to farmed insects (disease diagnosis) as well as those that can identify potentially harmful pathogens in insect food and feed for downstream applications (disease prevention).

## Data availability statement

The datasets presented in this study can be found in online repositories. The name of the repository and accession number can be found below: NCBI; PRJNA883751.

## Author contributions

DL, MO, BO were involved in all aspects of the project and manuscript preparation. All authors contributed to the article and approved the submitted version.

## Supplementary material

Additional File S1 Details from the analysis of sequence reads in Kraken2 (21). **(A)** Number of of reads from the collapsed colony (C-Adult, C-Diet, C-Frass), the new colony (N-Adult, N-Pupa, N-Diet), the recovered colony (R-Larva), and fresh diet (F-Diet) that were unclassified or classified by taxa rank. Classification at the phylum level were color coded: bacteria, yellow; archea, grey; eukaryote, green; and virus, orange. **(B)** Percentage of reads classified at the superkingdom rank.

Additional File S2 Species identified from reads in this study, with all reads associated with the collapsed colony (C-Adult, C-Diet, C-Frass, “Collapsed Colony Reads”) combined, and those associated with the new colony (N-Adult, N-Pupa, N-Diet, “New Colony Reads”) also combined, with a comparison of the number of reads from the collapsed and new colony reads (“Collapsed/New”).

Additional File S3 Reads with similarity to the genus *Bacillus*, with *B. thuringiensis* serovar *morrisoni* 4AA1 reads in yellow. Blast e-value, Blast top hit taxonomy name, and Blast hit accession are provided.
